# Lutetium ultraphosphate

**DOI:** 10.1107/S1600536808013032

**Published:** 2008-05-10

**Authors:** Karima Horchani-Naifer, Mokhtar Férid

**Affiliations:** a Unité de Recherches de Matériaux de Terres Rares, Centre National de Recherches en Sciences des Matériaux, BP 95 Hammam-Lif, 2050, Tunisia

## Abstract

The structure of the title compound, LuP_5_O_14_, comprises puckered eight-membered PO_4_ rings linked by the lutetium cations in a complex way, forming a three-dimensional framework. Each eight-membered phosphate ring shares a bridging tetra­hedron with each of four adjacent tetra­hedra, to form layers of PO_4_ tetra­hedra. These layers are *c*/2 in thickness and parallel to the *ab* plane. Each Lu ion is contained in one such layer, forming bonds to six O atoms in that layer and also to one O atom belonging to a tetra­hedron in each of the layers lying above and below it. The LuO_8_ polyhedra are isolated from one another, since they share no common atoms. The Lu ions lie on twofold axes (special position 4*e*) and the shortest Lu⋯Lu distance is 5.703 (1) Å.

## Related literature

For related literature, see: Durif (1971 ▶); Hong (1974 ▶); Hong & Pierce (1974 ▶). For the classification of ultraphosphates, see: Bagieu-Beucher & Tranqui (1970 ▶).

## Experimental

### 

#### Crystal data

LuP_5_O_14_
*M* *_r_* = 553.82
Monoclinic, 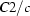

*a* = 12.8128 (14) Å
*b* = 12.6821 (13) Å
*c* = 12.3330 (13) Å
β = 91.295 (3)°
*V* = 2003.5 (4) Å^3^
*Z* = 8
Mo *K*α radiation
μ = 10.74 mm^−1^
*T* = 298 (2) K
0.20 × 0.19 × 0.18 mm

#### Data collection

Enraf–Nonius CAD-4 diffractometer
Absorption correction: multi-scan (*SADABS*; Sheldrick, 1996 ▶) *T*_min_ = 0.121, *T*_max_ = 0.145
10422 measured reflections
2912 independent reflections
2734 reflections with *I* > 2σ(*I*)
*R*_int_ = 0.031
2 standard reflections every 150 reflections intensity decay: 2%

#### Refinement

*R*[*F* ^2^ > 2σ(*F* ^2^)] = 0.019
*wR*(*F* ^2^) = 0.050
*S* = 1.08
2912 reflections
183 parameters
Δρ_max_ = 1.34 e Å^−3^
Δρ_min_ = −0.98 e Å^−3^

### 

Data collection: *CAD-4 EXPRESS* (Duisenberg, 1992 ▶; Enraf–Nonius, 1994 ▶; Macíček & Yordanov, 1992 ▶); cell refinement: *CAD-4 EXPRESS*; data reduction: *XCAD4* (Harms & Wocadlo, 1995 ▶); program(s) used to solve structure: *SHELXS97* (Sheldrick, 2008 ▶); program(s) used to refine structure: *SHELXL97* (Sheldrick, 2008 ▶); molecular graphics: *DIAMOND* (Brandenburg, 2001 ▶); software used to prepare material for publication: *SHELXL97*.

## Supplementary Material

Crystal structure: contains datablocks I, global. DOI: 10.1107/S1600536808013032/br2072sup1.cif

Structure factors: contains datablocks I. DOI: 10.1107/S1600536808013032/br2072Isup2.hkl

Additional supplementary materials:  crystallographic information; 3D view; checkCIF report

## Figures and Tables

**Fig. 1.**
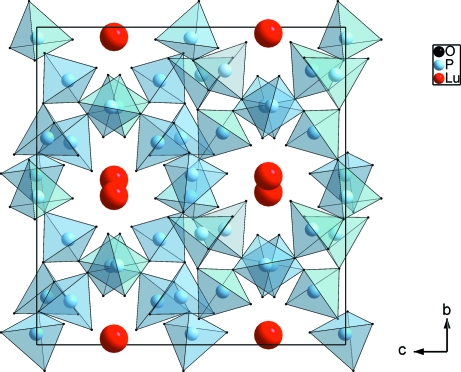
The structural arrangement of LuP5O14 along the a axis, showing tunnels in which the Lu ions are located.

**Fig. 2.**
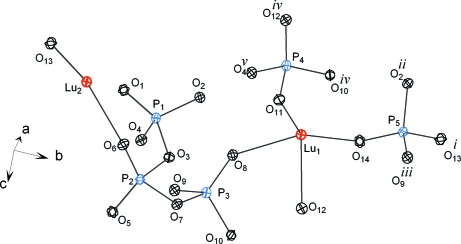
Projection of the ultraphosphate with anisotropic displacement parameters drawn at the 50% probability level. [Symmetry codes: (i) x, y+1, z ; (ii)-x+5/2, y+1/2, -z+3/2 ; (iii) x+1/2, y+1/2, z; (iv)x, -y+2, z-1/2 ; (v)x-1/2, -y+3/2, z-1/2.

**Fig. 3.**
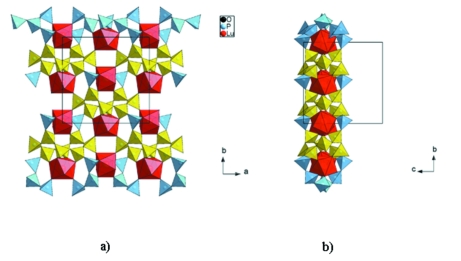
a) Projection of one layer showing LuO8 polyhedra and linkage of PO4 tetrahedra forming rings (yellow) in LuP5O14. b) Projection of one layer along the b axis with c/2 thickness.

## supplementary crystallographic information

### Comment

The structure of LuP_5_O_14_ is a type (II) rare-earth ultraphosphates as classified by Bagieu-Beucher & Tranqui (1970), since it crystallizes in the monoclinic system with space group *C*2/*c*. In this structure, the lutetium ion is surrounded by eight oxygen atoms that form distorted polyhedra. Each of the oxygen atoms in the LuO_8_ polyhedra are shared exclusively with PO_4_ tetrahedra to form a three-dimensional framework, which delimits interesting tunnels (Fig.1). The structure is built up from (PO_4_) tetrahedra (Fig.2) which are cross-linked by bridging O atoms, but these do not form helical ribbons, as in the NdP_5_O_14_ structure type (I) (Hong, 1974), and HoP_5_O_14_ structure type (III) (Durif, 1971).  The anion is being constructed from a succession of eight-membred rings interconnected through the ternary tetrahedra in a complex way (Fig.3a). The members of an individual ring are shown with yellow color. Each ring shares a bridging tetrahedron with each of four adjacent tetrahedra to form layers of PO_4_ tetrahedra, as illustrated in Fig.3b. These layers are about c/2 in thickness and parallel to the a-b plane. Each Lu ion is contained in one such layer, forming bonds to six oxygen in that layer and also to one oxygen belonging to a tetrahedron in each of the layers lying above and bolow it. the LuO_8_ polyhedra are isolated from one another, since they share no common atoms. The shortest Lu-Lu distances are 5.703. The LuP_5_O_14_ ultraphosphate is isostructural with YbP_5_O_14_ (Hong & Pierce, 1974).

### Experimental

Single crystal of LuP_5_O_14_ was prepared by flux method. At room temperature, 0.5 g of Lu_2_O_3_ were slowly added to 10 ml of phosphoric acid H_3_PO_4_ (85%). The mixture was then slowly heated to 673 K and kept at this temperature for seven days. colorless, crystals were separated from the excess phosphoric acid by washing the product in boiling water.

### Refinement

The highest peak and the deepest hole are located 0.79Å and 0.54 Å, respectively, from Lu1 and Lu2.

### Crystal data

**Table:**

LuP_5_O_14_	*F*_000_ = 2064
*M**_r_* = 553.82	*D*_x_ = 3.672 Mg m^−^^3^
Monoclinic, *C*2/*c*	Mo *K*α radiation λ = 0.71073 Å
Hall symbol: -C 2yc	Cell parameters from 7043 reflections
*a* = 12.8128 (14) Å	θ = 2.3–30.0º
*b* = 12.6821 (13) Å	µ = 10.74 mm^−^^1^
*c* = 12.3330 (13) Å	*T* = 298 (2) K
β = 91.295 (3)º	Prism, colorless
*V* = 2003.5 (4) Å^3^	0.20 × 0.19 × 0.18 mm
*Z* = 8	

### Data collection

**Table:**

Enraf–Nonius CAD-4 diffractometer	*R*_int_ = 0.031
Monochromator: graphite	θ_max_ = 30.0º
*T* = 298(2) K	θ_min_ = 2.3º
ω/2θ scans	*h* = −17→18
Absorption correction: multi-scan(SADABS; Sheldrick, 1996)	*k* = −17→17
*T*_min_ = 0.121, *T*_max_ = 0.145	*l* = −17→17
10422 measured reflections	2 standard reflections
2912 independent reflections	every 150 reflections
2734 reflections with *I* > 2σ(*I*)	intensity decay: 2%

### Refinement

**Table:**

Refinement on *F*^2^	Secondary atom site location: difference Fourier map
Least-squares matrix: full	*w* = 1/[σ^2^(*F*_o_^2^) + (0.028*P*)^2^ + 3.0307*P*] where *P* = (*F*_o_^2^ + 2*F*_c_^2^)/3
*R*[*F*^2^ > 2σ(*F*^2^)] = 0.019	(Δ/σ)_max_ = 0.003
*wR*(*F*^2^) = 0.050	Δρ_max_ = 1.34 e Å^−^^3^
*S* = 1.08	Δρ_min_ = −0.98 e Å^−^^3^
2912 reflections	Extinction correction: SHELXL97 (Sheldrick, 2008), Fc^*^=kFc[1+0.001xFc^2^λ^3^/sin(2θ)]^-1/4^
183 parameters	Extinction coefficient: 0.00637 (11)
Primary atom site location: structure-invariant direct methods	

### Special details

**Table:**

Geometry. All e.s.d.'s (except the e.s.d. in the dihedral angle between two l.s. planes) are estimated using the full covariance matrix. The cell e.s.d.'s are taken into account individually in the estimation of e.s.d.'s in distances, angles and torsion angles; correlations between e.s.d.'s in cell parameters are only used when they are defined by crystal symmetry. An approximate (isotropic) treatment of cell e.s.d.'s is used for estimating e.s.d.'s involving l.s. planes.
Refinement. Refinement of *F*^2^ against ALL reflections. The weighted *R*-factor *wR* and goodness of fit *S* are based on *F*^2^, conventional *R*-factors *R* are based on *F*, with *F* set to zero for negative *F*^2^. The threshold expression of *F*^2^ > σ(*F*^2^) is used only for calculating *R*-factors(gt) *etc*. and is not relevant to the choice of reflections for refinement. *R*-factors based on *F*^2^ are statistically about twice as large as those based on *F*, and *R*- factors based on ALL data will be even larger.

### Fractional atomic coordinates and isotropic or equivalent isotropic displacement parameters (Å^2^)

**Table:**

	*x*	*y*	*z*	*U*_iso_*/*U*_eq_	
Lu1	1.0000	1.019590 (11)	0.7500	0.00946 (6)	
Lu2	1.0000	0.469251 (11)	0.7500	0.00947 (6)	
P1	1.18263 (5)	0.63687 (5)	0.89095 (5)	0.01020 (13)	
P2	0.97504 (5)	0.65029 (5)	0.96672 (5)	0.01037 (13)	
P3	0.85012 (5)	0.83192 (5)	0.89783 (5)	0.01042 (13)	
P4	0.85469 (5)	0.96507 (5)	0.50182 (6)	0.01080 (14)	
P5	1.17542 (6)	1.24856 (5)	0.76032 (6)	0.01017 (14)	
O1	1.15332 (15)	0.54741 (15)	0.82204 (16)	0.0131 (4)	
O2	1.24259 (17)	0.72754 (16)	0.83475 (17)	0.0131 (4)	
O3	1.09081 (14)	0.69824 (15)	0.94396 (15)	0.0120 (3)	
O4	1.24970 (13)	0.60463 (17)	0.99342 (13)	0.0121 (4)	
O5	0.97503 (15)	0.59905 (15)	1.07328 (15)	0.0137 (4)	
O6	0.93961 (15)	0.59620 (15)	0.86627 (15)	0.0132 (4)	
O7	0.91291 (17)	0.75979 (14)	0.97911 (18)	0.0114 (4)	
O8	0.90954 (15)	0.87184 (15)	0.80717 (15)	0.0132 (4)	
O9	0.75333 (18)	0.76371 (15)	0.86473 (18)	0.0128 (4)	
O10	0.80606 (15)	0.91824 (14)	0.97493 (15)	0.0123 (4)	
O11	0.88893 (16)	0.96091 (15)	0.61582 (16)	0.0139 (4)	
O12	0.92416 (15)	1.06579 (15)	0.91286 (15)	0.0135 (4)	
O13	1.12590 (16)	0.34943 (15)	0.73083 (16)	0.0142 (4)	
O14	1.11367 (16)	1.15411 (15)	0.78473 (16)	0.0147 (4)	

### Atomic displacement parameters (Å^2^)

**Table:**

	*U*^11^	*U*^22^	*U*^33^	*U*^12^	*U*^13^	*U*^23^
Lu1	0.00912 (9)	0.00934 (9)	0.00992 (9)	0.000	−0.00025 (6)	0.000
Lu2	0.00918 (9)	0.00909 (8)	0.01011 (9)	0.000	−0.00038 (6)	0.000
P1	0.0100 (3)	0.0094 (3)	0.0112 (3)	0.0001 (2)	−0.0002 (2)	0.0002 (2)
P2	0.0100 (3)	0.0099 (3)	0.0112 (3)	0.0004 (2)	0.0001 (2)	0.0002 (2)
P3	0.0103 (3)	0.0097 (3)	0.0112 (3)	−0.0001 (2)	−0.0002 (2)	0.0000 (2)
P4	0.0108 (3)	0.0097 (3)	0.0119 (3)	−0.0006 (2)	−0.0003 (2)	0.0001 (2)
P5	0.0094 (3)	0.0099 (3)	0.0112 (3)	−0.0002 (2)	−0.0004 (3)	−0.0006 (2)
O1	0.0125 (9)	0.0125 (8)	0.0143 (9)	−0.0011 (7)	0.0008 (7)	−0.0012 (7)
O2	0.0142 (10)	0.0108 (8)	0.0144 (9)	−0.0013 (7)	0.0030 (7)	0.0002 (7)
O3	0.0099 (8)	0.0113 (8)	0.0149 (9)	0.0002 (7)	0.0013 (7)	−0.0004 (7)
O4	0.0115 (10)	0.0129 (9)	0.0118 (9)	0.0024 (6)	−0.0014 (7)	−0.0003 (6)
O5	0.0148 (9)	0.0127 (8)	0.0135 (9)	0.0004 (7)	−0.0001 (7)	0.0015 (7)
O6	0.0132 (9)	0.0135 (8)	0.0129 (9)	0.0018 (7)	−0.0009 (7)	−0.0010 (7)
O7	0.0107 (10)	0.0119 (8)	0.0116 (9)	0.0017 (6)	−0.0008 (7)	−0.0007 (6)
O8	0.0153 (9)	0.0122 (8)	0.0122 (8)	−0.0023 (7)	0.0012 (7)	−0.0001 (7)
O9	0.0119 (10)	0.0136 (8)	0.0129 (10)	−0.0016 (7)	−0.0012 (8)	0.0000 (7)
O10	0.0123 (9)	0.0098 (8)	0.0150 (9)	0.0007 (7)	0.0013 (7)	−0.0012 (7)
O11	0.0150 (10)	0.0142 (8)	0.0125 (9)	−0.0020 (7)	−0.0014 (7)	0.0002 (7)
O12	0.0143 (9)	0.0129 (8)	0.0135 (9)	−0.0007 (7)	0.0013 (7)	0.0002 (7)
O13	0.0148 (9)	0.0137 (8)	0.0142 (9)	0.0028 (7)	−0.0001 (7)	0.0002 (7)
O14	0.0156 (9)	0.0131 (8)	0.0151 (9)	−0.0044 (7)	−0.0009 (7)	0.0002 (7)

### Geometric parameters (Å, °)

**Table:**

Lu1—O14	2.2775 (19)	P2—O7	1.6097 (19)
Lu1—O14^i^	2.2775 (19)	P2—O3	1.633 (2)
Lu1—O11	2.283 (2)	P3—O8	1.458 (2)
Lu1—O11^i^	2.283 (2)	P3—O9	1.559 (2)
Lu1—O8	2.3213 (19)	P3—O10	1.5638 (19)
Lu1—O8^i^	2.3213 (19)	P3—O7	1.566 (2)
Lu1—O12	2.3260 (19)	P4—O11	1.464 (2)
Lu1—O12^i^	2.3260 (19)	P4—O12^iv^	1.481 (2)
Lu2—O13^i^	2.2326 (19)	P4—O4^v^	1.6111 (19)
Lu2—O13	2.2326 (19)	P4—O10^iv^	1.637 (2)
Lu2—O6	2.3016 (19)	P5—O13^vi^	1.470 (2)
Lu2—O6^i^	2.3016 (19)	P5—O14	1.470 (2)
Lu2—O1	2.356 (2)	P5—O2^vii^	1.614 (2)
Lu2—O1^i^	2.356 (2)	P5—O9^viii^	1.623 (2)
Lu2—O5^ii^	2.3604 (19)	O2—P5^ix^	1.614 (2)
Lu2—O5^iii^	2.3604 (19)	O4—P4^x^	1.6111 (19)
P1—O1	1.462 (2)	O5—Lu2^iii^	2.3604 (19)
P1—O2	1.555 (2)	O9—P5^xi^	1.623 (2)
P1—O3	1.5659 (19)	O10—P4^xii^	1.637 (2)
P1—O4	1.5665 (18)	O12—P4^xii^	1.481 (2)
P2—O5	1.466 (2)	O13—P5^xiii^	1.470 (2)
P2—O6	1.479 (2)		
			
O14—Lu1—O14^i^	82.98 (10)	O13—Lu2—O5^iii^	76.40 (7)
O14—Lu1—O11	140.00 (7)	O6—Lu2—O5^iii^	73.85 (7)
O14^i^—Lu1—O11	73.88 (7)	O6^i^—Lu2—O5^iii^	142.33 (7)
O14—Lu1—O11^i^	73.88 (7)	O1—Lu2—O5^iii^	73.25 (7)
O14^i^—Lu1—O11^i^	140.00 (7)	O1^i^—Lu2—O5^iii^	126.65 (7)
O11—Lu1—O11^i^	141.95 (10)	O5^ii^—Lu2—O5^iii^	136.94 (9)
O14—Lu1—O8	150.35 (7)	O1—P1—O2	115.98 (12)
O14^i^—Lu1—O8	109.89 (7)	O1—P1—O3	116.30 (11)
O11—Lu1—O8	69.49 (7)	O2—P1—O3	101.66 (11)
O11^i^—Lu1—O8	79.86 (7)	O1—P1—O4	113.30 (12)
O14—Lu1—O8^i^	109.89 (7)	O2—P1—O4	106.56 (12)
O14^i^—Lu1—O8^i^	150.35 (7)	O3—P1—O4	101.33 (10)
O11—Lu1—O8^i^	79.86 (7)	O5—P2—O6	122.63 (12)
O11^i^—Lu1—O8^i^	69.49 (7)	O5—P2—O7	106.70 (11)
O8—Lu1—O8^i^	72.36 (10)	O6—P2—O7	109.67 (11)
O14—Lu1—O12	85.80 (7)	O5—P2—O3	109.73 (11)
O14^i^—Lu1—O12	72.29 (7)	O6—P2—O3	106.95 (11)
O11—Lu1—O12	116.25 (7)	O7—P2—O3	98.52 (11)
O11^i^—Lu1—O12	73.85 (7)	O8—P3—O9	114.72 (12)
O8—Lu1—O12	73.73 (7)	O8—P3—O10	115.17 (11)
O8^i^—Lu1—O12	133.38 (7)	O9—P3—O10	104.58 (12)
O14—Lu1—O12^i^	72.29 (7)	O8—P3—O7	115.10 (12)
O14^i^—Lu1—O12^i^	85.80 (7)	O9—P3—O7	103.75 (11)
O11—Lu1—O12^i^	73.85 (7)	O10—P3—O7	101.94 (11)
O11^i^—Lu1—O12^i^	116.25 (7)	O11—P4—O12^iv^	121.99 (12)
O8—Lu1—O12^i^	133.38 (7)	O11—P4—O4^v^	105.88 (11)
O8^i^—Lu1—O12^i^	73.73 (7)	O12^iv^—P4—O4^v^	108.80 (11)
O12—Lu1—O12^i^	150.82 (9)	O11—P4—O10^iv^	109.37 (11)
O13^i^—Lu2—O13	94.22 (10)	O12^iv^—P4—O10^iv^	108.74 (11)
O13^i^—Lu2—O6	98.99 (7)	O4^v^—P4—O10^iv^	99.76 (11)
O13—Lu2—O6	142.75 (7)	O13^vi^—P5—O14	121.90 (13)
O13^i^—Lu2—O6^i^	142.75 (7)	O13^vi^—P5—O2^vii^	104.38 (11)
O13—Lu2—O6^i^	98.99 (7)	O14—P5—O2^vii^	112.10 (11)
O6—Lu2—O6^i^	91.22 (10)	O13^vi^—P5—O9^viii^	110.34 (11)
O13^i^—Lu2—O1	147.63 (7)	O14—P5—O9^viii^	104.96 (11)
O13—Lu2—O1	74.22 (7)	O2^vii^—P5—O9^viii^	101.37 (12)
O6—Lu2—O1	76.11 (7)	P1—O1—Lu2	138.32 (12)
O6^i^—Lu2—O1	69.60 (7)	P1—O2—P5^ix^	141.48 (14)
O13^i^—Lu2—O1^i^	74.22 (7)	P1—O3—P2	125.49 (12)
O13—Lu2—O1^i^	147.63 (7)	P1—O4—P4^x^	129.58 (12)
O6—Lu2—O1^i^	69.60 (7)	P2—O5—Lu2^iii^	170.97 (13)
O6^i^—Lu2—O1^i^	76.11 (7)	P2—O6—Lu2	138.19 (12)
O1—Lu2—O1^i^	130.25 (10)	P3—O7—P2	133.72 (14)
O13^i^—Lu2—O5^ii^	76.40 (7)	P3—O8—Lu1	141.86 (12)
O13—Lu2—O5^ii^	74.67 (7)	P3—O9—P5^xi^	138.16 (14)
O6—Lu2—O5^ii^	142.33 (7)	P3—O10—P4^xii^	127.96 (13)
O6^i^—Lu2—O5^ii^	73.85 (7)	P4—O11—Lu1	148.50 (12)
O1—Lu2—O5^ii^	126.65 (7)	P4^xii^—O12—Lu1	147.46 (12)
O1^i^—Lu2—O5^ii^	73.25 (7)	P5^xiii^—O13—Lu2	151.04 (13)
O13^i^—Lu2—O5^iii^	74.67 (7)	P5—O14—Lu1	156.41 (13)

Symmetry codes: (i) −*x*+2, *y*, −*z*+3/2; (ii) *x*, −*y*+1, *z*−1/2; (iii) −*x*+2, −*y*+1, −*z*+2; (iv) *x*, −*y*+2, *z*−1/2; (v) *x*−1/2, −*y*+3/2, *z*−1/2; (vi) *x*, *y*+1, *z*; (vii) −*x*+5/2, *y*+1/2, −*z*+3/2; (viii) *x*+1/2, *y*+1/2, *z*; (ix) −*x*+5/2, *y*−1/2, −*z*+3/2; (x) *x*+1/2, −*y*+3/2, *z*+1/2; (xi) *x*−1/2, *y*−1/2, *z*; (xii) *x*, −*y*+2, *z*+1/2; (xiii) *x*, *y*−1, *z*.
